# Exploring a Digital Health Solution to Collect and Manage Health-Related Needs for Patients Who Undergo Complex Surgery: Mixed Methods Study

**DOI:** 10.2196/77995

**Published:** 2025-12-08

**Authors:** Naleef Fareed, Humza Asgher, Diamantis Tsilimigras, Odysseas P Chatzipanagiotou, Giovanni Catalano, Bridget Hartwell, Kathleen Bolton, Timothy M Pawlik

**Affiliations:** 1 Department of Biomedical Informatics, College of Medicine, The Ohio State University, 370 West 9th Avenue, Columbus, OH, 43210, United States, 1 614 366 0283; 2 Department of Surgery, College of Medicine, The Ohio State University, Columbus, OH, United States; 3 James Surgical Oncology Department, The Ohio State University Wexner Medical Center, Columbus, OH, United States

**Keywords:** health needs, social needs, surgery, digital health, electronic health record data

## Abstract

**Background:**

Patients who undergo complex surgery (eg, esophagectomy and liver resection) often experience a substantial burden of health-related needs (medical, social, and behavioral health). A digital solution could facilitate the collection and resolution of health-related needs by care team members for these patients. A digital solution may facilitate adherence to a clear treatment plan and concomitantly reduce surgical complications and readmissions.

**Objective:**

The aim of the study is to establish gaps in the collection and management of health-related needs and identify a set of user specifications for a digital solution to collect and resolve health-related needs for patients who undergo complex surgery.

**Methods:**

We applied the double diamond framework and organized the study into two sequential phases: (1) qualitative methods to discover the perspectives of patients and care team members on health-related needs and (2) participatory design sessions to gain feedback and sentiment about ideal features of a digital solution. Both phases were conducted between December 2023 and March 2025. We supplemented both phases with analysis of electronic health record (EHR) data for patients who underwent complex surgery at our academic medical center.

**Results:**

Extensive themes emerged from interviews with patients (n=20) and care team members (n=24), capturing their health-related and surgical experiences as well as desired features for a digital solution. Our swimlane diagram demonstrated four critical gaps in workflow: (1) heterogeneity in the approach to screening, monitoring, and managing health-related needs; (2) patients felt uncomfortable reporting health-related needs, particularly behavioral and social needs, to their care team; (3) lack of access to referral resources to resolve needs; and (4) the need for a closed-loop intervention for patients and care team members. A subset of participants from phase 1 (n=5 patients and n=9 care team members) provided feedback on preferred features, drawing from digital tools currently available in the EHR at our academic medical center. Among the 3 existing EHR tools tested, there were variations in how patients and care team members felt about their potential use. Participants also provided extensive feedback for preferred components (eg, goals and active plans) that should be available in an existing or custom digital solution to manage health-related needs. Findings from the qualitative interviews and design sessions were corroborated with documentation in the EHR.

**Conclusions:**

Digital solutions could provide a streamlined approach for the collection and management of health-related needs in surgery, with the goal of addressing unmet needs and improving patient activation. This approach is critical to ensure that patients, especially patients who undergo complex surgery, have positive health outcomes. We identified preferences for specific features in a proposed digital solution based on our systematic assessment that will inform future work.

## Introduction

The evidence on how to best collect and manage health-related needs is inadequate. Health care organizations in the United States are especially faced with challenges in implementing policies that increase requirements in reporting health-related needs [1-4]. Furthermore, the integration of health-related social needs with medical and behavioral needs represents a system-level effort to recognize the importance of whole-person health as a mechanism for transforming health care quality [5,6]. The collection, integration, and management of health-related needs (ie, medical, social, and behavioral) can be complicated and time-intensive, especially with social needs requiring substantial resources to resolve unmet needs [7]. Screening rates and documentation of social needs have greatly varied across clinics, and hospital systems require effective collaboration with referral organizations, such as community-based organizations (CBOs), to effectively address these needs [8].

Studies on health-related needs, particularly social needs, have been conducted in internal medicine, pediatrics, and outpatient settings [9,10]; however, effective screening in surgical care remains poorly defined. Social needs have been linked to complications and mortality during and soon after a surgical episode [11]. Patients with unaddressed social needs are twice as likely to seek care in the emergency department [12], which may disrupt treatment goals, negatively affect recovery from surgery, and contribute to readmissions. Despite this, integration of medical, social, and behavioral needs to provide holistic care remains a significant challenge in surgery, like many other clinical settings, and data from real-world implementation are limited [13,14].

Digital solutions provide a promising and comprehensive approach to improve the collection of health-related needs, particularly social needs. Digital solutions can collect real-time data [15] and enable real-time management of needs that can enhance the surgical experience [16]. Timely management of needs with digital technologies can help patients recover earlier after surgery and shorten their hospital stay [16]. Digital solutions allow personalized content for each patient [17], which can further support an individualized approach to address specific patient needs. Novel solutions have been proposed in the literature to collect and refer patients to local resources using various digital health platforms [18,19].

To enhance the collection and management of health-related needs, it is essential to learn from the perspectives of both patients and care team members. The perspectives of these key stakeholders can help identify barriers and facilitators, ultimately refining interventions to promote acceptance of such tools and optimize both treatment goal management and health-related needs that can impact the goals. Extant literature indicates that screening of health-related needs, particularly social needs, is less prevalent in specialist visits compared with primary care settings, as screening for these factors may be challenging to integrate into specialized workflows [20,21]. Thus, implementing a standardized approach, which may be grounded in a technological solution, may facilitate screening across different specialties. To bridge the gap in the literature, we conducted a mixed methods study on both care team members and patients to assess convergent and divergent themes focused on health-related needs screening and management in surgical settings at our academic medical center (AMC). The objective of this study was to identify feature specifications for a digital solution that can transform the existing workflow at our AMC to improve the management of patients who undergo complex surgery. Specifically, we sought (1) to establish problems and gaps in the collection, integration, and management of health-related needs in the current treatment approach for patients who undergo complex surgery at our AMC and (2) to define desired specifications for a digital solution, informed by feedback referencing digital tools currently available in the existing electronic health record (EHR) system. Our study is particularly important, given the inherent challenges of collecting evidence on sensitive topics within historically marginalized and vulnerable communities.

## Methods

### The Double Diamond Framework

In this study, we followed the “double diamond” framework for our user-centered design approach [22]. The double diamond framework can be applied to design and develop a digital solution based on formative evidence. The framework allowed us to define problems identified by care team members and patients in the collection and management of health-related needs, suggest digital solutions for participants, and gather user perceptions regarding design and usability. The framework represents a structured design thinking process consisting of 5 phases: diagnosis, discovery, definition, development, and delivery. In our study, the diagnosis, discovery, and definition phases guided the exploratory work, which included qualitative interviews, literature review, and quantitative data analysis to understand the problem space and identify unmet needs. Findings from these activities were synthesized using swimlane diagrams to refine the patient journey and prioritize critical gaps. The development and delivery phases then informed our solution-oriented activities, including prototyping and testing with end users, to ensure that proposed solutions were desirable and further modifiable to their feedback upon validation. Both phases were conducted between December 2023 and March 2025. We explain our work from each of the phases below.

### Diagnose

Our research team first performed a scoping review of the literature for major gaps related to the collection and management of health-related needs in surgery, with a particular focus on social needs. Our study focused on experiences with health-related needs and preferences for digital solutions to report such needs. We reviewed various peer-reviewed papers to identify gaps in the current literature, including formative work for this study [23]. We also reviewed our AMC EHR progress notes for patients who underwent complex surgery in 2021, specifically the structured and unstructured fields where information on health-related needs has been historically documented. We calculated how many hours case managers and social workers spent on resolving health-related needs, as recorded in a productivity flow sheet, and assessed how these needs were captured in the EHR.

### Phase I: Discover

Our research team conducted one-on-one interviews with care team members and patients from the department of surgery at our AMC. The department of surgery performs approximately 15,000 operations a year and has a broad catchment area across the state. In these interviews, we aimed to identify current barriers and facilitators of the collection and management of health-related needs information. Interviews examined three main areas of interest: (1) current workflows for preoperative, perioperative, and postoperative care; (2) current workflows and processes for the collection of health-related needs information with a particular emphasis on social needs; and (3) participants’ preferences regarding the design of digital solutions to manage unmet needs. See MultimediaAppendices 1  2 for patient and care team interview guides, respectively.

### Study Sample

#### Care Team Members

Care team members were recruited from our AMC department of surgery. Inclusion criteria included attending physicians, resident physicians, social workers, case managers, and advanced practice providers who work in the department of surgery and are at least 18 years of age. Eligible care team members, identified by the department of surgery research team as providers of care for the patient population, were invited by email. The invitation included a brief overview of the study, a description of participant activities, and a time commitment. The final sample of care team members represents a convenience sample.

#### Patients

Patients were recruited from our AMC department of surgery. Inclusion criteria were English-speaking patients of at least 18 years of age who underwent complex surgery (eg, cardiovascular, bariatric, or gastrointestinal procedures) requiring at least 1 overnight stay. Patient recruitment was stratified by racioethnicity and neighborhood Ohio Opportunity Index (OOI) score (lesser opportunity and most opportunity), and eligible patients were recruited. We oversampled patients by racioethnicity based on evidence that indicates resource-limited populations have less access to quality care compared to other individuals in surgery [24,25]. One of these studies also noted that marginalized communities were more likely to experience adverse surgical outcomes [25]. In addition to racioethnicity, neighborhood characteristics may also play a role in surgical outcomes and patient experience. Racioethnicity was measured using self-reported information in the EHR: Hispanic, non-Hispanic Black, non-Hispanic White, and non-Hispanic other. An individual’s residence can affect their air quality, water quality, and transportation access, which critically impacts their health [26]. Thus, we stratified patients by OOI, which is an area deprivation–based measure. The OOI is comprised of social and economic opportunities (ie, income, employment, transportation, and education) present across the state of Ohio. OOI scores range from 1 to 100. Higher OOI scores indicate greater opportunity in a neighborhood. Further details regarding the development and use of OOI can be found in the study by Fareed et al [27]. In our study, we divided OOI scores into septiles and used the septiles to define 2 groups: lower opportunity represented by neighborhoods with OOI scores that are in septiles 1, 2, 3, and 4; and higher opportunity represented by neighborhoods with OOI scores higher than the fourth septile. Participants’ racioethnicity and OOI information are included with quotes to provide additional context.

### Semistructured Interviews

Eligible participants were identified by a research team member and subsequently recruited by email or phone. Patient participants, more specifically, were first identified with the EHR-based age, inpatient stay, language preference, and specific *International Classification of Diseases, Ninth Revision, Clinical Modification* (ICD-9-CM) and *International Classification of Diseases, Tenth Revision, Procedural Codes* (ICD-10-PCS; please see Table 1 for codes). One-on-one interviews were audio-recorded and lasted approximately 60 minutes. The research team conducted the interviews using semistructured guides. The interview guides were designed by the research team, pilot-tested on care team members and mock patients, and refined based on feedback from the pilot. The interview guide for care team members consisted of 4 sections: care team member background (roles and care workflow), medical care (typical course of treatment and challenges to medical and behavioral recommendations), collection and management of social needs information, and desired features in a digital solution for managing unmet needs. The interview guide for patients included 4 sections: patient background (surgery procedure), medical care, information on social needs, and desired features in digital solutions for managing unmet needs. Questions for the guide were based on prior research and guidance from experts in the research team [28-30]. See MultimediaAppendices 1  2 for patient and care team interview guides, respectively.

**Table 1.** *International Classification of Diseases, Ninth Revision, Clinical Modification* (ICD-9-CM) and *International Classification of Diseases, Tenth Revision, Procedural Codes* (ICD-10-PCS) used to screen patient eligibility for the study in the electronic health record.

Procedure	ICD-9-CM codes	ICD-10-PCS codes
Coronary artery bypass grafting	361*	0210*; 0211*; 0212*; 0213*
Aortic valve replacement	352*	02RF*; X2RF*
Mitral valve replacement	352*	02RG*
Abdominal aortic aneurysm repair	3505; 3834; 3844; 3864; 3925; 397*; 3978; 3505	02RF*; 0410*; 04500ZZ; 04B00ZZ; 04R0*; 04U0*; 04V0*; X2RF332
Carotid endarterectomy	3812.0	03CH*; 03CJ*; 03CK*; 03CL*; 03CM*; 03CN*
Carotid stent	63.0	037H*; 037J*; 037K*; 037L*
Peripheral vascular intervention	1756; 3950; 3990; 55; 60	027P*; 027Q*; 027R*; 027S*; 027T*; 027V*; 027W*; 027X*; 02CP3ZZ; 02CQ3ZZ; 02CR3ZZ; 02CS3ZZ; 02CT3ZZ; 02CV3ZZ; 02CW3ZZ; 02CX3ZZ; 0370*; 0371*; 0372*; 0373*; 0374*; 0375*; 0376*; 0377*; 0378*; 0379*; 037A*; 037B*; 037C*; 037D*; 037F*; 037G*; 037H*; 037J*; 037K*; 037L*; 037M*; 037N*; 037P*; 037Q*; 037R*; 037S*; 037T*; 037U*; 037V*; 037Y*; 03C0*; 03C1*; 03C2*; 03C3*; 03C4*; 03C5*; 03C6*; 03C7*; 03C8*; 03C9*; 03CA*; 03CB*; 03CC*; 03CD*; 03CF*; 03CR*; 03CS*; 03CT*; 03CU*; 03CV*; 03CY*; 0470*; 0471*; 0472*; 0473*; 0474*; 0475*; 0476*; 0477*; 0478*; 0479*; 047A*; 047B*; 047C*; 047D*; 047E*; 047F*; 047H*; 047J*; 047K*; 047L*; 047M*; 047N*; 047P*; 047Q*; 047R*; 047S*; 047T*; 047U*; 047V*; 047W*; 047Y*; 04C0*; 04C1*; 04C2*; 04C3*; 04C4*; 04C5*; 04C6*; 04C7*; 04C8*; 04C9*; 04CA*; 04CB*; 04CC*; 04CD*; 04CE*; 04CF*; 04CH*; 04CJ*; 04CK*; 04CL*; 04CM*; 04CN*; 04CP*; 04CQ*; 04CR*; 04CS*; 04CT*; 04CU*; 04CV*; 04CW*; 04CY*; 0570*; 0571*; 0573*; 0574*; 0575*; 0576*; 0577*; 0578*; 0579*; 057A*; 057B*; 057C*; 057D*; 057F*; 057G*; 057H*; 057L*; 057M*; 057N*; 057P*; 057Q*; 057R*; 057S*; 057T*; 057V*; 057Y*; 0670*; 0671*; 0672*; 0673*; 0674*; 0675*; 0676*; 0677*; 0678*; 0679*; 067B*; 067C*; 067D*; 067F*; 067G*; 067H*; 067J*; 067M*; 067N*; 067P*; 067Q*; 067R*; 067S*; 067T*; 067V*; 067Y*; NoPCS
Amputation	841*	0Y620ZZ; 0Y630ZZ; 0Y640ZZ; 0Y670ZZ; 0Y680ZZ; 0Y6C*; 0Y6D*; 0Y6F0ZZ; 0Y6G0ZZ; 0Y6H*; 0Y6J*; 0Y6M*; 0Y6N*
Esophagectomy	424*; 4399	0DB1*; 0DB2*; 0DB3*; 0DB5*; 0DT1*; 0DT2*; 0DT3*; 0DT5*; 0DT6*
Pancreatic resection	5222; 525*; 526; 527	0F5G*; 0FBG0ZZ; 0FTG0ZZ
Colectomy	173*; 457*; 458*	0DBE*; 0DBF*; 0DBG*; 0DBH*; 0DBK*; 0DBL*; 0DBM*; 0DBN*; 0DTE*; 0DTF*; 0DTG*; 0DTH*; 0DTK*; 0DTL*; 0DTM*; 0DTN*
Rectal resection	484*; 485*; 486*	0DBP*; 0DTP*
Bariatric	438*; 443*; 445; 4468; 449*	0D16*; 0DB6*; 0DH6*; 0DL6*; 0DL7*; 0DQ6*; 0DV64CZ; 0DW6*
Gastrectomy	434*; 435; 436; 437; 438*; 4399	0D56*; 0D57*; 0DB4*; 0DB6*; 0DB7*; 0DT4*; 0DT6*; 0DT7*
Liver resection	5022; 503	0FB0*; 0FB1*; 0FB2*; 0FT1*; 0FT2*
Lung resection	322*; 323*; 324*; 325*; 326; 329	01B30ZZ; 01BL0ZZ; 0B5C*; 0B5D*; 0B5F*; 0B5G*; 0B5H*; 0B5J*; 0B5K*; 0B5L*; 0B5M*; 0BBC*; 0BBD*; 0BBF*; 0BBG*; 0BBH*; 0BBJ*; 0BBK*; 0BBL*; 0BBM*; 0BTC*; 0BTD*; 0BTF*; 0BTG*; 0BTH*; 0BTJ*; 0BTK*; 0BTL*; 0BTM*
Incisional hernia repair	535*; 536*	0WQF*; 0WUF*
Cholecystectomy	512*	0F54*; 0FB4*; 0FT4*
Appendectomy	470*; 471*	0DTJ*

Interviews were conducted virtually on Zoom (Zoom Video Communications) or in-person at our AMC. All interviews were transcribed verbatim using the online platform Zoom. Primary interviewers were female in their early 20s and male in their late 20s and 30s. Secondary interviewers included a male in their early 20s. All interviewers were English-speaking. The research team discussed after each interview whether new themes emerged and achieved agreement for the themes of our study to ascertain thematic saturation. To monitor saturation, the research team met weekly to review the interviews completed during the prior week. Interviewers compared any newly observed concepts, noted in field notes, against the existing codebook. New codes were documented, and existing codes were refined as needed based on these discussions. Saturation was considered achieved when interviews yielded no new codes and only reinforced existing themes. To ensure adequacy, we conducted 3 additional interviews per group, which confirmed the recurrence and depth of the identified themes. Codebook available from the author (NF) upon request.

We interviewed 44 (20 care team members and 24 patients) participants. Phase I: 8 of 28 (29%) care team members could not participate due to scheduling conflicts or nonavailability. In total, 27 of 51 (53%) patients did not participate because they either refused or did not show up to the interview after accepting our invitation. Phase II: 3 care team members could not participate due to scheduling conflicts or nonavailability. In total, 14 of 19 (70%) patients either refused or did not show up to the interview after accepting our invitation.

### Ethical Considerations

All study activities were approved by our AMC institutional review board (The Ohio State University; #2023H0301). Participants provided informed consent to participate in the study, participation was voluntary, and the participants could withdraw at any time. All participants were consented to the study by a trained research team member before the interview. Each nonphysician care team member received a US $50 gift card after completion of both interview phases (ie, discovery and design sessions). The collected data were anonymized and deidentified.

### Define

We used a thematic analysis approach to organize care team members and patient interviews through open coding. Interviews were analyzed abductively: deductively to categorize results and inductively to discover emerging themes and subthemes. Initially, 2 team members (HA and a trained research assistant) coded 3 transcripts independently for each group to ensure that our codebooks were appropriate, and codes were refined to ensure consistency if needed.

These team members staggered the coding process for the remaining transcripts, with both members independently coding all manuscripts. Team members met weekly to review the consistency of coding and agreement about code definitions. Team members also discussed the recurrence of themes to ensure that data saturation was achieved. A third, senior researcher independently reviewed the coded transcripts and made final decisions when necessary. Two researchers selected exemplary quotes that reflected emerging themes from the transcripts. To maintain rigor, we performed peer debriefing and member checking with care team members to confirm our identified themes. We also documented our decisions during the data collection and analysis stages. Microsoft Word was used to manually code and analyze the transcripts. For the analysis of phase I data, the size and scope of the dataset made Microsoft Word both practical and sufficient. The relatively modest number of documents and codes enabled us to manage the data effectively without compromising rigor. To ensure transparency and credibility comparable to computer-aided qualitative data analysis software, we implemented several procedures: maintaining a versioned and iteratively refined codebook, documenting the evolution of coding decisions through memoing in Microsoft Excel, and preserving an audit trail of changes across coding rounds using Microsoft SharePoint. We followed COREQ (Consolidated Criteria for Reporting Qualitative Research) guidelines in reporting our findings (Multimedia Appendix 3) [31].

### Phase II

#### Develop

The goal of phase II was to gather initial feedback on phase I findings and catalog desirable features for tools to manage health-related social needs. We recognize the importance of representativeness within the surgical population at our AMC and nationally; this was not the goal of our phase II activities, and achieving this goal with vulnerable populations for research on sensitive topics is challenging. Rather, our main priority was to maximize the perspectives gathered from these communities. We asked participants about how to enhance patient activation levels in preparation for their surgery and recovery. Patient activation is an evidence-based concept that represents an individual’s confidence, self-management skills, and self-efficacy [32]. Given our emphasis on whole-person health, we chose sample systems and tools that encompass health-related needs: medical needs (eg, hypertension and diabetes), behavioral needs (eg, diet, alcohol, and tobacco use), and social needs (eg, food and transportation) to ensure a holistic and integrated approach to patient care during the surgery journey. To not assume that a patient is solely responsible for addressing their needs, we augmented our framework with social cognitive theory (SCT) and goal-setting theory (GST) [33,34], both of which assume the need for a supportive environment within which a patient can successfully feel activated and achieve their treatment goals. Our team used 3 Epic-based products to gain initial feedback on available designs that can encompass a digital solution to our identified issues from phase I. We chose to use Epic-based tools because of the familiarity of the system at our AMC among participants and to generate insights that can be generalized to other settings, given the ubiquity of Epic among health care systems in the United States. None of these tools were widely deployed at our AMC at the time of our study or were considered as part of the AMC goal of providing whole-person care for patients. We centered our design proposition on patient activation because the challenges a patient faces regarding their confidence, self-management skills, and self-efficacy are associated with a successful complex surgery outcome that includes readmission, emergency department visit, and postoperative complications. Prior literature has suggested that patients undergoing complex surgery experience low levels of confidence, self-management, and efficacy, particularly among patients faced with unmet needs [35]. We further augment the need to enhance a patient’s activation levels with support from their care team that can be informed by other behavioral theories: SCT and GST. During our phase II sessions, we used these concepts to center our discussion around the use of the various Epic tools. This approach enabled participants to identify both desirable and less desirable features in tools to help achieve higher patient activation levels, which they recognized was an important aspect of achieving improved health.

The 3 tools that were shown during the design sessions were Longitudinal Plan of Care (LPOC), Compass Rose (CR), and EpicCare Link. LPOC provides a detailed summary of a patient’s treatment plan, goals, and active plans, which aligns with the development of patient knowledge and skills using our patient activation framework. This dashboard could be seen by care team members at our AMC to manage and guide patients on their goals. The main features on the LPOC dashboard are care coordination notes, allergy and problem lists, social determinants of health (SDoH) wheel, goals, and active plans. There are 4 main features of the LPOC patient view: goals, to-do list, medications, and care team information. CR is a care coordination tool that allows the care team to make referrals for patients with social and behavioral needs to community organizations (eg, food pantry). CR offers features that allow the user to monitor steps necessary to achieve a specific goal (eg, complete an application for a referral program), which can help improve patient skills and confidence using our patient activation framework. The main sections of CR are program-specific targets, timeline, care team information, and a sidebar that describes the programs the patient is enrolled in, social needs the patient has, and different types of risk scores (ie, admission to the emergency department, general risk score, and low patient engagement risk) [36]. EpicCare Link is a platform that allows CBOs to access care management tools. CBOs can access an Epic portal and message a care team member at the AMC, add to the patient’s LPOC, and help address patient care gaps. CBOs can update a care coordination note and update steps related to a specific patient’s goal that is subsequently integrated into the AMC EHR. The main features of the EpicCare Link dashboard are care coordination notes, allergy and problem lists, SDoH section, goals, care team, and recent visits.

#### Deliver

We conducted design interviews with a subset of participants from phase I of our study. Care team members were shown an example action plan for a patient who needed to make sure they had a high-protein diet for 6 weeks after surgery. This action plan was conceptually organized with a specific, measurable, achievable, relevant, and time-bound (SMART) goal. SMART goals can guide patients to achieve a clear and tailored health goal [37]. Care team members were then shown 3 screenshots of digital tools: LPOC, CR, and EpicCare Link depending on their role at the AMC. The clinical care team was primarily shown LPOC and CR. The nonclinical care team was primarily shown CR and EpicCare Link. Patients were shown the same example of the action plan as the care team and the patient view of LPOC. Care team and patient participants were also asked about whether the example prototypes would improve patient activation levels. See Multimedia Appendix 4 for a sample SMART goal and patient action plan.

To analyze the design interviews, we conducted a sentiment analysis based on features. Codes were created based on specific features of the digital tools that were presented during the design sessions. A researcher manually coded all phase II transcripts using MAXQDA and subsequently conducted the sentiment analysis in MAXQDA to categorize quotes as positive, slightly positive, neutral, slightly negative, or negative sentiment. This approach allowed our team to determine the nuances of user perceptions and attitudes on the digital tools and identify specific features that enhanced whole-person care for patients who undergo complex surgery. The MAXQDA sentiment analysis feature uses the SentiWordNet 3.0 lexicon for English [38]. We selected the stop word list option on MAXQDA to ignore words that do not have any sentiment. After this step, 2 members of the research team manually reviewed the MAXQDA codes and overrode miscoded quotes. Another researcher reviewed the manual and MAXQDA codes to ensure that the coding was accurate and consistent. A senior researcher selected exemplary quotes for each sentiment. We evaluated the digital tools based on sentiment and cataloged the desired features and recommendations provided by participants for a final set of desired user specifications in a whole-person–based digital solution.

## Results

### Participant Characteristics

We recruited 20 care team members and 24 patients. Details about care team member and patient characteristics are noted in Tables2  3, respectively. Care team members consisted of 35% (7/20) physicians, 20% (4/20) resident physicians, 20% (4/20) case managers, 15% (3/20) advanced practice providers, and 10% (2/20) licensed social workers. Among patients who were interviewed, a total of 8% (2/24) were Hispanic, 46% (11/24) were non-Hispanic Black, 42% (10/24) were non-Hispanic White, and 4% (1/24) were non-Hispanic other. Among the 16 patients living in a lower opportunity neighborhood, a total of 13% (2/16) were identified as Hispanic, 44% (7/16) identified as non-Hispanic Black, 38% (6/16) identified as non-Hispanic White, and 6% (1/16) identified as non-Hispanic other. Among the 8 patients living in a neighborhood with a higher opportunity, 50% (4/8) were identified as non-Hispanic Black, and 50% (4/8) identified as non-Hispanic White. Approximately 63% (15/24) underwent gastrointestinal or benign surgery, 8% (2/24) underwent gastrointestinal or oncology surgery, 13% (3/24) underwent general or hernia surgery, 4% (1/24) underwent general or soft tissue surgery, 8% (2/24) underwent cardiovascular surgery, and 4% (1/24) underwent nongastrointestinal or oncology surgery. In phase II, we conducted digital sessions with a subset of participants from phase I. We interviewed a total of 9 care team members: 33% (3/9) case managers, 22% (2/9) advanced practice providers, 22% (2/9) social workers, and 22% (2/9) physicians. We interviewed a total of 5 patients, of whom 3 patients reported their sex as female and 2 reported their sex as male. The average age of the patient participants was 58 (SD 17) years. In total, 60% (3/5) of patients underwent gastrointestinal surgery, and 40% (2/5) underwent general surgery. Among patients interviewed, 20% (1/5) were non-Hispanic Black, and 80% (4/5) were non-Hispanic White. Among the patients who identified as non-Hispanic White, 50% (2/4) live in a neighborhood with a lower opportunity. The patient identified as non-Hispanic Black lives in a neighborhood with a lower opportunity.

**Table 2.** Care team member participant background information.

Characteristic	Distribution (n=20)
Role, n (%)	
Attending physician	7 (35)
Resident physician	4 (20)
Case manager	4 (20)
Advanced practice provider	3 (15)
Social worker	2 (10)
Sex, n (%)	
Female	12 (60)
Male	8 (40)
Age (years), mean (SD)	41 (12)

**Table 3.** Patient participant background information.

Characteristic	Distribution (n=24)
Racioethnicity, n (%)	
Hispanic	2 (8)
Non-Hispanic Black	11 (46)
Non-Hispanic White	10 (42)
Non-Hispanic other	1 (4)
Ohio Opportunity Index (OOI)^a^, n (%)	
Lesser opportunity	16 (67)
Most opportunity	8 (33)
Sex, n (%)	
Male	13 (54)
Female	11 (46)
Age (years), mean (SD)	58 (17)
Surgery type, n (%)	
Gastrointestinal or benign	15 (63)
Gastrointestinal or oncology	2 (8)
General or hernia	3 (13)
General or soft tissue	1 (4)
Cardiovascular	2 (8)
Nongastrointestinal or oncology	1 (4)

a OOI categories were defined as follows: lesser opportunity OOI, which includes septiles 1 through 4; and most opportunity OOI, which includes septiles 5 through 7.

### Diagnosis Phase

#### Overview

Patients who undergo surgery and their care team members struggled to effectively collect and manage health-related needs, particularly social needs. Care team members faced challenges such as time constraints and the inability to identify appropriate resources for their patients, which can delay patients’ ability to achieve clinical treatment goals. Patients, moreover, were overwhelmed with social needs while trying to balance these needs with preparation and recovery from surgery.

#### Problem

Care team members faced challenges with addressing health-related needs, particularly social needs, due to time constraints [20]. Care team members felt that they did not have the time and resources to successfully address these needs [39]. Case managers and social workers who recorded their activity time in the EHR productivity flow sheet spent an average of approximately 6 (SD 15) hours per patient addressing social needs. Some care team members offered a resource packet to patients. Social worker 2 explained:

Some people think that we can like get them an apartment which, unfortunately we can’t. ... Unfortunately, so, if they’re homeless, we have like a homeless hotline. We give them and then for the housing resources we have lists of like section 8, housing and apartments. That we give to the patient and kind of just say I’m not sure like what’s available for these departments ...

Care team members shared that common challenges for patients to meet medical goals are a lack of access to resources. Case manager 1 explained:

I would say the big ones tend to be like lack of support, whether that’s if they don’t have a lot of support people, or they don’t have transportation or, you know, lack of resources. If you know their lower income or are not insured or under insured ... more kind of generally on the medical side, would just be general compliance ...

There are several challenges that prevent patients from meeting their medical goals. These challenges differ depending on the population [40]. Physician 1 mentioned:

... there’s social determinants of health and kind of health literacy components social support, you know, and family members or loved ones who are able to encourage adherence to plans and treatment strategies. Certainly, in the bariatric population there’s a lot of metabolic component to their adherence to you know, durable weight management and diet patterns. Obviously, there’s financial problems ... I think travel can be challenging for our patient population ...

For example, patients may experience financial issues, which can be a major challenge in following care team recommendations and may delay medical treatment [41]. Patient 23 (male patient, in their 40s, general or soft tissue surgery, non-Hispanic Black, lesser opportunity neighborhood) shared:

I’m working for Uber. You know. I’m driving. I don’t know whether I will qualify to collect disability. You know what I mean? Because that’s it’s not a small surgery. It’s a big surgery that would take like maybe three months to stay home, and I have family to feed. I got kids. I got mortgage to pay. So it’s crazy. I have to plan that. It’s not something I can just come do it. you know, doesn’t work like that ...

### Digital Solution Tools

Digital health advancements provide an opportunity to better address health-related needs in the hospital system. A closed-loop approach has been proposed to address these needs, with communication, management, and resolution between the patient and care team. We used 3 Epic tools that could be fully implemented at our AMC to potentially align with this approach. We coded user feedback during the design interviews and organized quotes based on sentiment. For the care team, EpicCare Link had the highest number of quotes (25/80) with positive or slightly positive sentiments. LPOC had 17 of 65 quotes, and CR had 17 of 79 quotes coded as positive or slightly positive sentiment. The patient view of LPOC had 8 of 44 quotes with positive or slightly positive sentiment. Care team members saw the value in incorporating these digital tools into their current workflow. Overall, the care team understood the importance of most sections in each digital tool. However, there were suggestions to add customizable features that yielded a slightly negative or negative sentiment such as adding more information or adding a drop-down option for a section. CR had the most quotes (19/79) with slightly negative or negative sentiment. EpicCare Link had the second-highest number of quotes (17/80) with slightly negative or negative sentiment. LPOC had a total of 14 of 65 quotes with slightly negative or negative sentiment. The patient view of LPOC had 5 of 44 quotes with slightly negative or negative sentiment.

Patients overall liked the idea of having a digital tool designed for their health-related goals on the patient view of LPOC. There were recommendations made that focused on rewording sections to encourage patients to be more engaged in their active plan. There were 21 of 49 quotes coded with slightly positive or positive sentiment. There were 23 of 49 quotes coded with slightly negative or negative sentiment for the patient view of LPOC.

### Phase I: Discover

#### Overview

When we asked care team members about health-related needs, many expressed the need for local resources that address social needs and for a streamlined process to collect and manage this information. When we asked patients about health-related needs, they expressed social needs challenges and emphasized the importance of closing the loop to resolve these issues. Our interviews captured barriers, facilitators, experiences, and future considerations with health-related needs.

#### Perceived Impacts of Managing Health-Related Needs

##### Benefits

Both care team members and patients understood the value of the collection and management of health-related needs information. Care team members emphasized that health-related needs can affect the patient’s surgical outcomes and their overall health. Case manager 4 mentioned:

Honestly, it can make or break the success of the overall care because you can say: Hey, we’re gonna do this as a doctor. We’re gonna do this, this and this cause that. What that’s what needs to be done. If they don’t have food to heal that wound. It’s not gonna heal right if they don’t have clean water. If they don’t have transportation or insurance information or somebody that can help them. Then they might have a setback like I said, in the perfect world everything I listed out for you on that piece of paper or that care plan is smooth. That never happens ...

Patients felt that the collection of health-related needs information can help them better prepare for their surgery and recovery process. Patients believed that feedback on health-related needs from the care team is important and feels like they are being cared for. One patient shared that the collection and management of health-related needs information are necessary to effectively capture and assess patients’ health. Patient 7 (male patient, in their 60s, gastrointestinal or oncology, non-Hispanic White, lesser opportunity) shared:

So inside, it’s a necessity. You’re not trying your best. If you’re not doing that. Okay, you know you’re shorting yourself. All you’re doing is you’re shorting yourself. You don’t give them the full story.

##### Barriers

Care team members believe that some patients may not be comfortable or honest in sharing health-related needs information. Patients may share information but do not want the information to be on their record (eg, domestic violence). Social worker 2 shared:

If patients are forthcoming. Sometimes they’re embarrassed. They don’t want to talk about it, which I totally understand. But it just depends. Like, I said, some patients there are patients that need a lot of things, but they just don’t want to talk to me. I think sometimes the social worker can leave a bad taste into some people’s mouths. They might have had interactions with social workers in the past, and it been kind of negative ...

A patient shared during their interview that they would not feel comfortable sharing health-related needs information, particularly social needs, with their care team. Patient 19 (male patient, in their 30s, gastrointestinal or benign, Hispanic, lesser opportunity) explained:

[TRANSLATOR SPEAKING] So, he was basically saying that like he didn’t get like, why, it would be important to like share your social needs with the doctor, because he says like, even though he would have problems, he wouldn’t specifically communicate it with doctor ...

Another patient felt like a burden to the care team with their health-related needs and, as a result, did not want the care team’s help with addressing them.

One patient highlighted that the health-related needs information is being collected but not managed properly. There is a lack of feedback to patients to address these needs. Patient 20 (male patient, in their 60s, gastrointestinal or benign, non-Hispanic Black, most opportunity) shared:

I think a lot of the information goes in, and that’s [IT] and it just stops.

It was mentioned that patients with lower socioeconomic status may have difficulties that the care team must consider during the collection and management of health-related needs processes. Another patient emphasized that the collection of health-related needs information was redundant and questioned why this information is not synthesized in one place for the care team to reference. Patient 7 (male patient, in their 60s, gastrointestinal or oncology, non-Hispanic White, lesser opportunity) noted:

You know, I’ve been asked a lot of the same questions over and over again. So I was wondering why it wasn’t all documented in my charts, so everybody could see the whole story. My story, and as thorough as I find my charts to be and I really like that program. I found it a little bit redundant, but at the same time I was very patient about answering all the questions when it was asking.

### Current Collection and Management of Health-Related Needs

#### Awareness

Care team members were aware of the collection of health-related needs information, and it was not consistently implemented. Resident physician 1 stated:

Yes and no [PATIENTS REPORT HEALTH-RELATED NEEDS]. I mean, I think if we ask if we ask about it, and we ask about it because they’re usually relevant to their condition. Yeah, I think they certainly do report it. You know, unless we’re asking about it. I don’t think it’s like freely brought up often.

Those who do not collect information may informally ask their patients questions relevant to their practice (ie, asks a patient whether they have social support for recovery after an operation). Physician 5 shared:

So they don’t frequently offer it. No, it’s not something that is routinely part of our assessment, really. And I always ask people the things that I do always ask, are you gonna be able? Are you gonna have somebody that’s gonna be able to help you out afterwards? But I don’t go into the nitty, nitty gritty of the social situation. I’ve had that bite me a couple of times.

The Epic SDoH Wheel is available to care team members at our AMC. We found that many care team members were not familiar with this feature. However, care team members may use other Epic functions for noting health-related needs (ie, Inbasket messages or progress notes in Epic). Advanced practice provider 1 shared:

I’m not sure what that is the social determinant of health wheel. I’m not sure. I mean, I use the in basket all the time. And you know, for calls and messaging like my chart messages. We use that pretty much every single day. But I’m not sure what the social wheel is.

Many patients could not recall if health-related needs information was collected before, during, or after their surgery. Some patients recalled being asked once a description of social information was provided in the interview. Other patients were unfamiliar with health-related needs. Patient 6 (male patient, in their 60s, gastrointestinal or benign, non-Hispanic White, most opportunity) explained:

Like I said. You know I was there a couple of 3 days . I said I need a shower, and they’re very obliged, and they got me hooked up. We wouldn’t, you know, have shower and everything. So I mean That’s social needs, cause I was feeling pretty funny, and I, you know. But they hooked me up.

#### Roles

Some patients could not recall the names of care team members who collected their health-related needs. Patients reported that case managers were the care team members most involved in the collection of health-related needs. This aligns with what care team members shared. Care team members who collect health-related needs ask for both medical and social-related needs. Case managers and social workers are the specified team members responsible for the collection of this information. Social worker 2 noted:

So for all patients that like have any needs like homelessness, addiction, medicine like issues of transportation, etc. We’ll get a consult from the nurse to do like our social work assessment, or I’ll just do a chart review and kind of see that their needs and so I’ll complete our social work assessment, just asking some questions about the social determinants of health and then giving a really big resource packet ...

#### Documentation Process

A psychosocial assessment—a structured tool—is used by case managers at our AMC to collect some health-related needs information. This information is placed in the EHR progress notes for all care team members to review; however, only a few review it outside of case management and social work. Care team members who provide oncology care use the Oncology Distress Survey to collect health-related needs information. Advanced practice provider 2 shared:

There is the [ONCOLOGY] distress screen ... So that gets sent to all of the oncology patients and then if they fill it out on my chart and it flags, it comes back and the flow is that you’re supposed to call the patient. If there are flag responses and discuss referrals.

Other care team members evaluate where the patient currently is before surgery regarding health-related needs (eg, employment and financial issues) to assess perioperative risk and use this information to develop a surgical plan. They have their own questionnaire and ask questions verbally. The collection process varied greatly among patients. One patient mentioned that they had a small talk with a care team member. Other patients recalled a questionnaire that focused on food and housing. Patient 21 (male patient, in their 50s, gastrointestinal or oncology, non-Hispanic Black, most opportunity) noted:

It was a series of questions that they would ask on the screen. There were three different people that come in and ask the same question one after the other.

Some care team members expressed that they are uncomfortable asking patients direct questions regarding health-related needs. Physician 1 mentioned:

I sort of intentionally do not ask them a lot of other social things that can be a little off putting to patients, I think, who are simply requesting a surgical procedure be performed. And a lot of times, I think. If they want to volunteer information. Talk about information I’m happy to ...

This collection process may be seen as redundant. Another patient mentioned that the care team member documented health-related needs but did not follow up or use that information. Patient 20 (male patient, in their 60s, gastrointestinal or benign, non-Hispanic Black, most opportunity) shared:

“No, they came to solve me, and I gave them the same information. But and they supposedly well, but not, there’s no follow up ... They just asked it and annotated it. And that was it.

#### Monitoring Health-Related Needs

Some care team members have a unique way of monitoring health-related needs. For instance, in the note history, a care team member will place a plus sign to indicate that they had a conversation regarding a sensitive topic the patient brought up. They can then refer to the conversation based on what the care team member recalls.

Care team members have noticed an increase in documentation of health-related needs. As a result, these care team members make the effort to review patient health-related needs. Case manager 3 explained:

Within the chart, the EHR chart. We’ll go and review, like what information’s available from progress notes and things that the social workers and outpatient center, or whoever else. Because I’ve noticed lately that more providers are being involved in that and asking those questions. So I will review those kind of things when the patients are coming in, so that I know kind of what we’re facing and what’s been done.

Some patients expressed that if they had a health-related needs issue, they would feel comfortable enough to let their care team know. Patient 5 (female patient, in their 30s, gastrointestinal or benign, Hispanic, lesser opportunity) noted:

Yeah, I would at least call up either by calling them in or sending a message through like a portal.

#### Basic Social Needs

We defined a basic social need as a need that affects the patient’s daily life but does not require a comprehensive solution from a financial perspective (eg, food, transportation, and medication assistance) [42,43]. Transportation was a basic social need that was mentioned most frequently. Food insecurity and medication assistance were the second and third most frequently mentioned basic needs among patients, respectively. Transportation may be the highest because many of the AMC patients travel from long distances to the AMC for care. Social worker 2 shared:

Yeah, I would say transportation to honestly a big one that I didn’t, that we run into a lot. But the patients have rights home can’t drive when they leave here. So I think transportation is a big one, and just making sure that patients have a safe ride home.

Some patients live far from the AMC, which can contribute to transportation issues. One patient wished they had known about reporting transportation issues to the care team. Patient 14 (female patient, in their 50s, gastrointestinal or benign, non-Hispanic Black, lesser opportunity) shared:

That would be excellent [RECEIVE TRANSPORTATION ASSISTANCE], because if I could have reported transportation problems, my son could have been at work today, and their bus could pick me up, brought me here took me up. That would have been perfect.

#### Complex Social Needs

We defined a complex social need as a need that requires extensive assistance with different factors of patients’ daily lives. This involves a more comprehensive solution from a financial perspective (eg, housing, finances, employment, education, social support and home care, air quality, and domestic violence) [42,43]. Based on our interviews, social support and home care were the complex needs with the highest frequency. Social support and home care include patients who may live alone or lack friends or family to help with their care and recovery.

Among patients, the most frequently mentioned complex need was social support and home care. Some patients needed someone at home to change their dressing after surgery. One patient shared that they lived alone and had a family member stay with her to make sure someone was near her during recovery. Patient 2 (female patient, in their 70s, gastrointestinal or benign, non-Hispanic White, lesser opportunity) shared:

I really don’t have anyone that lives with me ... Yeah [I had someone stay with me after surgery], just actually, the first night that I came home. Yeah, my cousin was here, but she had a dog, which the dog irritated me, so I said, I don’t think I need you ...

**Figure 1.**
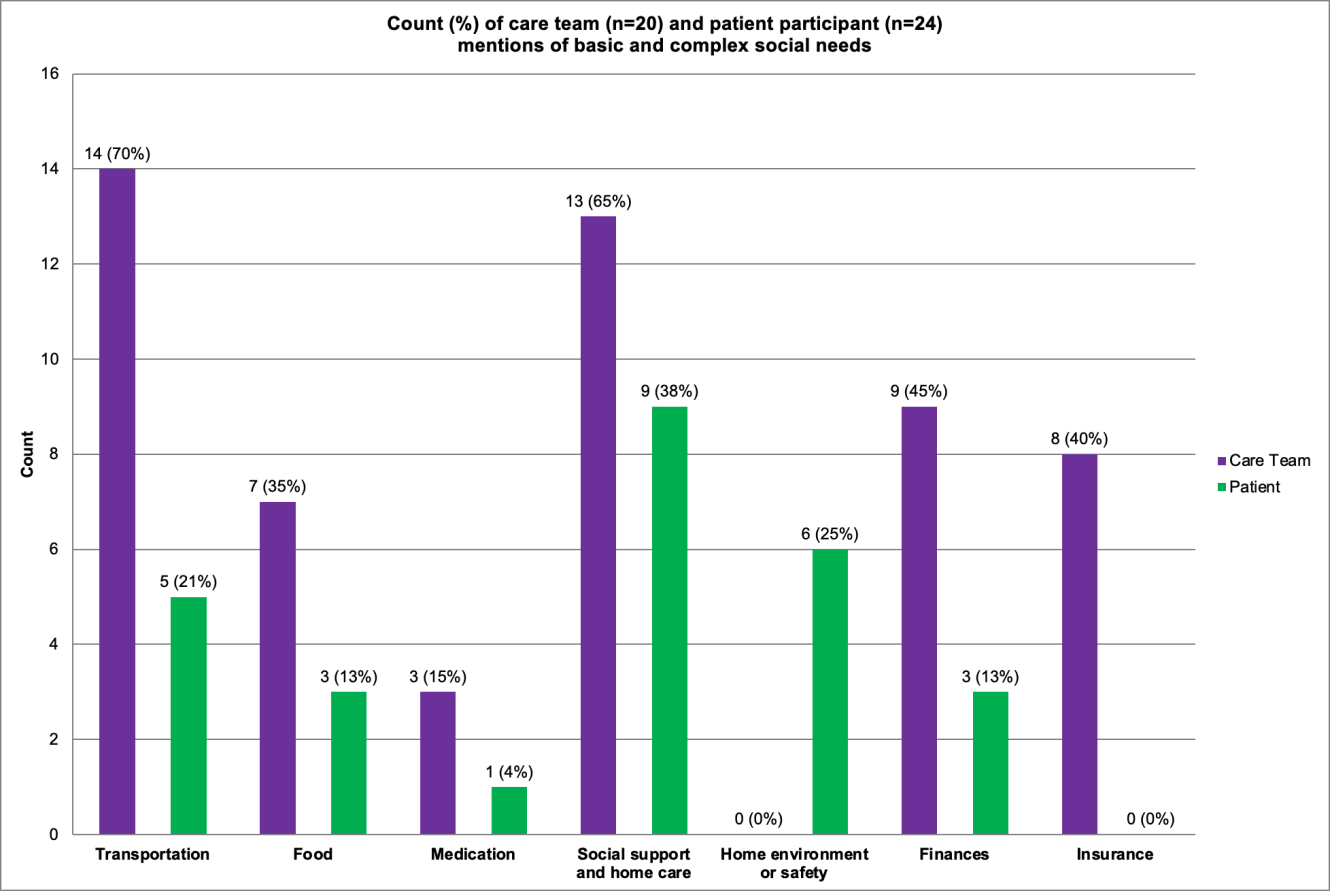
Frequency of the top basic and complex social needs reported by both care team members and patient participants during the research study. Percentages reported by participant type (purple: care team and green: patient).

Home environment or home safety and financial concerns were the second and third highest complex needs, respectively. Figure 1 provides a breakdown of basic and complex social needs for care team members and patients. Among patients who discussed social needs when we specifically inquired about basic and complex needs, 75% (9/12) lived in lower opportunity neighborhoods, 56% (5/9) of whom identified as non-Hispanic Black. We also found that 50% (12/24) of patients who did not discuss any social needs during our interview lived in lower opportunity neighborhoods, and 50% (6/12) of whom identified with a marginalized community.

#### Resolution of Health-Related Needs

One care team member specifically shared that they did not know where to refer patients to address a social need. Care team members focused on medical needs may have the patient connect with a care team member centered on social needs (ie, social services, social worker, and case manager). Some care team members noted that they are not familiar with local resources in their patients’ communities, especially rural areas. Physician 2 noted:

Usually what I do is I loop in social work or the PCRM [PATIENT CARE RESOURCE MANAGER] to help with that? Because although a lot of my patients are here over in the [DEIDENTIFIED] community. A number of them come from farther away, and I don’t know the resources for the cities that are like two and three hours away ...

Recently, the AMC implemented the UNITE US, a platform integrated with Epic intended to provide automated access to resources. Some care team members expressed concerns about “closing the loop” with patients to ensure that they have indeed received the resources they were referred to due to challenges such as inadequate community capacity and linkages for referrals made through UNITE US. Physician 5 explained:

When a patient comes to me and tells me that their husband is unwilling to help them with their recovery, because he’s out drinking every night. What? What do I? What I do with that actually had that happen recently? And I asked her and made sure she was safe. There was no concern for abuse or anything like that. But yes, she felt really, really lost, and really unsupported, and what I can’t, what can I do about that? So asking people to report is one step, but being able to support is a separate and I think more important ...

Patients experienced some challenges in resolving health-related needs. Some care team members did not follow up with a patient on a health insurance–related concern. Patient 20 (male, in their 60s, gastrointestinal or benign, non-Hispanic Black, most opportunity) shared:

... I wanted to get my wife reimbursed ... So they said, call them. I’ve asked that question four or five times through my care team. I’ve never got a response to that question of who should I go through? Is there a telephone number, and I know this is not the first time that the care team and the hospital has heard that you know how what agency reimburses for that I know some. I guess it’s called Medicaid ... if a person has a question. Don’t just take down information, follow up either in my chart, which is a very expedient way, my chart, or send a person a message, or you have their numbers. Give them a call.

Another patient needed transportation for an appointment, and the care team provided transportation assistance to the patient. A patient did not expect health-related needs to be addressed during the collection process. The patient thought that the care team wanted a baseline of health-related needs history for patients. See Multimedia Appendix 5 for exemplary quotes from care team members (Table S1 in Multimedia Appendix 5) and patients (Table S2 in Multimedia Appendix 5).

### Future Approaches to Collect and Manage Health-Related Needs: Define

**Figure 2.**
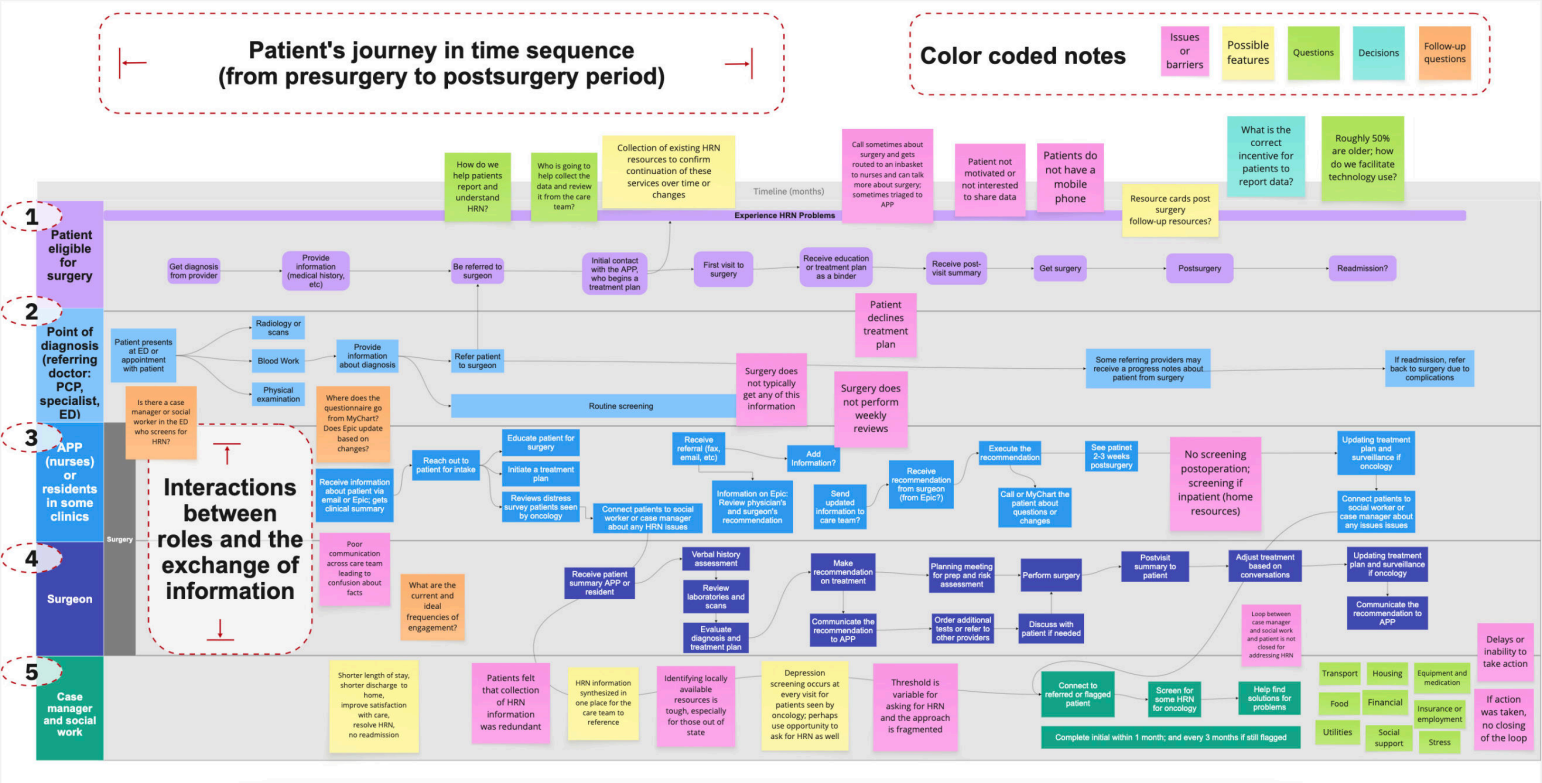
Swimlane of “as-is” clinical workflow for patients who undergo complex surgery at AMC. The figure illustrates time-sequenced interactions across five roles: (1) patient, (2) point of diagnosis, (3) APPs or residents, (4) surgeon, and (5) case manager or social work in the management of a patient who undergoes complex surgery at AMC, based on insights provided by research participants. AMC: academic medical center; APP: advanced practice provider; ED: emergency department; HRN: health-related need; PCP: primary care physician.

We further analyzed the formative interviews using swimlane diagrams. A swimlane diagram allows us to visualize the interactions and workflows in the AMC. We used the “as-is” swimlane diagram to map out the current workflows for a surgical patient (Figure 2). We included workflows for the surgical patient, referring physician, advanced practice providers, surgeons, case managers, and social workers. Each role is indicated in a different color. The arrows and lines from one note to another represent the flow of interaction. Each note is color-coded to describe barriers identified by participants, possible digital solution features, questions, decisions to be made, and follow-up questions. We identified four critical gaps in our “as-is” swimlane diagram: (1) heterogeneity in the approach to screening, monitoring, and managing health-related needs; (2) patients may feel uncomfortable reporting health-related needs, particularly behavioral and social needs, to their care team; (3) lack of access to referral resources to resolve needs; and (4) the need for a closed-loop intervention for patients and care team members. As previously noted, the care team spent almost 6 hours per patient on the coordination of health-related social needs based on our assessment of patient EHR charts from 2021. We also identified some health-related needs collection in the EHR among 55% of patients. Most of the recorded needs were focused on tobacco and alcohol use as well as depression. There were some data recorded in a psychosocial assessment structured field under clinical progress notes, and some information was documented under social history.

To address these critical gaps, we centered our design proposition on patient activation because the challenges a patient faces with regard to their confidence, self-management skills, and self-efficacy are associated with a successful complex surgery outcome. We further augment the need to enhance a patient’s activation levels with support from their care team that can be informed by other behavioral theories: SCT and GST [33,34]. SCT posits that the successful performance of a behavior is dependent on an individual’s capabilities, cognitive influences, and environmental factors. These influences are organized in three domains: (1) knowledge and beliefs, (2) skills, and (3) self-efficacy [33]. GST proposes that motivating individuals to enhance performance and improve self-efficacy requires goal clarity, commitment, feedback, and task complexity [44,45]. Patients may not see the relevance or trust the care team regarding health-related needs and could be unaware of how addressing unmet needs can help them achieve their treatment goals. Based on GST, for example, the surgery treatment plan can be developed as a playbook to ensure that the patient achieves their treatment goals. For patients to be motivated to achieve their goals, SCT is essential to provide the necessary information and skills for patients to take an active role in managing their health while being cognizant of their health-related needs, which can be acquired with support from the care team and the community.

### Phase II

#### Develop

**Figure 3.**
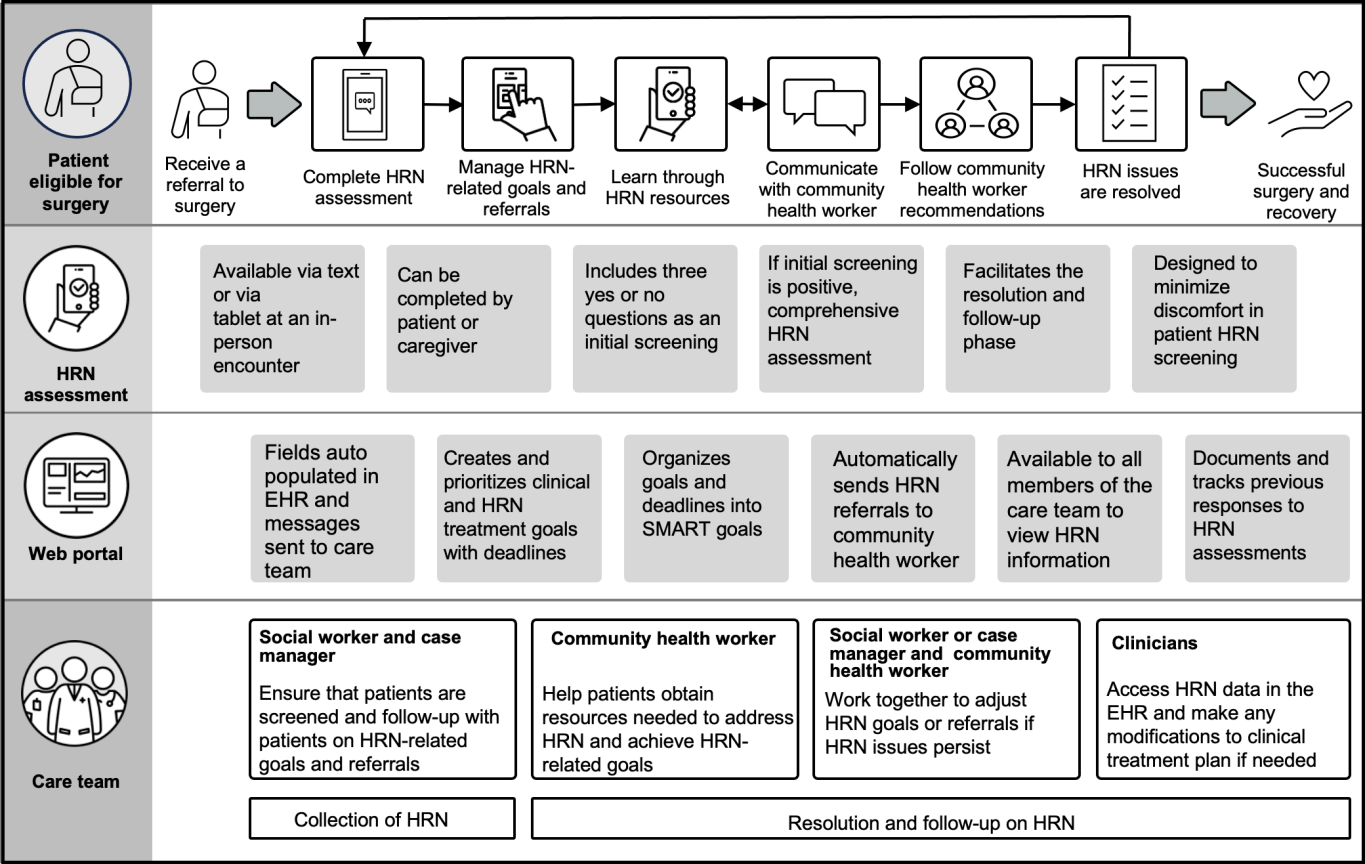
“To-be” swimlane of clinical workflow for patients who undergo complex surgery at AMC. The figure outlines a new process for identifying and addressing health-related needs among patients who undergo complex surgery at AMC based on the insights provided by study participants. AMC: academic medical center; EHR: electronic health record; HRN: health-related need; SMART: specific, measurable, achievable, relevant, and time-bound.

Based on our results from phase I, the research team created a “to-be” swimlane diagram (Figure 3) that addresses the identified major gaps of the current workflow. Given that health-related needs screening can involve sensitive topics such as housing or financial troubles [29], we implemented measures in our intervention to mitigate potential discomfort and help foster trust. Patients can report health-related needs using a web app—accessible through a smartphone or the web, both independently or with the help of a nurse navigator. This standardized approach enables the collection of health-related needs from a broad panel of patients. Once patients complete their health-related needs assessment, they will be automatically referred to resources to address their needs—triaged by the nurse navigator to specific care team members based on the type of need. Care team members will then follow up on patients to ensure that health-related needs have been met, with the intent to close the loop between the patient and the resource need. In addition to ethical handling of patient data, other important safeguards to respect patient privacy that can be considered include (1) access controls (eg, based on roles and need to know basis), (2) workflow protocols (eg, referral to trained staff and safety procedures), and (3) user autonomy (eg, giving patients the right controls to disclose what they want, whom they want to share this information, and when they want to receive care).

#### Deliver

In addition to an ideal workflow, we also gathered desired features for a digital solution that would be embedded in this workflow. Participants were asked questions during the design interviews about whether Epic tools would improve patient activation levels. Most digital tools had positive sentiment among the care team participants. LPOC, LPOC patient view, and CR had more positive or slightly positive sentiment than negative or slightly negative sentiment. There was an equal number of quotes coded as positive or slightly positive and negative or slightly negative for EpicCare Link.

Sentiments varied depending on the digital tool shown and the tool-specific features. The care team had the most positive or slightly positive sentiment (29%) for the active plans feature of the LPOC. The SDoH Wheel had the most negative or slightly negative sentiment (43%). Three care team members expressed that they would prefer a list or tab section. The ability to print the LPOC was the feature with the most positive or slightly positive sentiment (50%) in the LPOC patient view. The to-do list in the LPOC patient view had the highest number of coded quotes with negative or slightly negative sentiment (60%). The care team wanted to ensure that the to-do list items were tailored to the upcoming surgery and recovery. Referrals, social drivers, and the sidebar in the CR dashboard had the most positive or slightly positive sentiment (24%). The CR targets had the most negative or slightly negative sentiment (42%). Care team members wanted more information than what was shown to better understand patients’ needs specific to programmatic goals. The SDoH feature in the EpicCare Link view had the most quotes with positive or slightly positive sentiment (24%). However, the SDoH and goals features had the most quotes with negative or slightly negative sentiment (24%). Care team members wanted to ensure that the SDoH covered all social needs domains (eg, financial and employment). The goals section needed to be clearer on the specific goal and progress toward that goal.

Patients overall felt that the LPOC patient view would improve patient activation levels and be helpful in preparing and recovering from the surgery. Based on the patients, the care team, goals, and to-do list features had the most coded quotes with slightly positive or positive sentiment (35%) for the LPOC patient view. The goals feature had the most quotes with slightly negative or negative sentiment (40%). Examples of recommendations from patients included a progress on goals feature and providing alternative approaches to achieve goals. See Tables S3 and S4 in Multimedia Appendix 5 for desired user specifications by feature and technology from the care team and patient design interviews, respectively.

## Discussion

### Principal Findings

There were several convergent themes among patients and care team members. Health-related needs collection processes varied greatly for patients and care team members, supporting the idea that there is currently no established or widely used tool to accurately identify, integrate, and address health-related needs in the perioperative period at our AMC. A few care team members had a process for screening health-related needs, but most did not. Many care team members found the screening tools in the EHR (ie, Epic SDoH Wheel) not helpful and did not use the tool. Based on the literature, even with screening tools in the EHR, there is a variation in the consistency of documentation [46], which aligns with our formative interviews and our chart review. There were various ways patients remembered sharing health-related needs with their care team. Screening surveys and small talk were also mentioned in the formative interviews, which aligns with another previously published study [28].

Both care team members and patients understood the benefits of the collection of health-related needs. Care team members emphasized that the collection of health-related needs can better prepare patients and themselves to handle such needs. Care team members mentioned that some patients may be reluctant to report health-related needs, which also aligned with our phase I patient interview data and existing evidence [28]. Both groups mentioned that the collection and management process needs to be streamlined to improve effectiveness. Moreover, research highlights the importance of fostering trust as an important antecedent to successful patient engagement with respect to addressing health-related needs [42]. Both care team members and patients shared experiences with resolved health-related needs and unresolved needs. They also highlighted the importance of a closed-loop process when addressing health-related needs. Since awareness of treatment goals has been found to improve health outcomes [47], integrating goals for health-related needs in a closed-loop process could ensure that these patients’ needs are screened and addressed effectively.

The collection of social needs is particularly challenging. Both patients and care team members noted the same top 3 basic social needs. Transportation was the most frequently mentioned basic need for both groups. Both groups identified social support and home care as the top complex social need. Social support has been linked to improved health outcomes, with the most noted basic and complex social needs mentioned in our interviews aligned with the literature [11]. At the national level, our analysis of the National Cancer Institute’s Health Information National Trends Survey indicated that food, transportation, and housing account for >50% of reported social needs, which align with some of the top social needs identified in our formative interviews. Based on our experience working with communities that experience significant unmet needs, we have identified important safeguards that should be considered. In addition to ethical handling of the data (ie, Health Insurance Portability and Accountability Act [HIPAA] compliance and encryption), other safeguards that can be considered include (1) access controls (eg, based on roles and need to know basis), (2) workflow protocols (eg, referral to trained staff and safety procedures), and (3) user autonomy (eg, giving patients the right controls to disclose the type of information that they want, with whom they want to share this information, and when they want to receive care).

There were also divergent themes between participants. Patients felt that the documentation process was redundant. Patients could not recall many details regarding the collection process, or some patients lacked familiarity with the concept of health-related needs, contributing to the disconnect. Some care team members expressed that they felt uncomfortable discussing sensitive health-related needs information (ie, finances) with patients. There were also different perspectives surrounding resources and referrals. Some patients were not aware of the resources offered by their care team. Care team members expressed that the current resources are inadequate, which is a national issue, given the need for multisectoral partnerships and capacity building in various disinvested communities.

### Design Requirements and Refinements Needed to Meet End-User Preferences and Needs

Based on the formative interviews, we selected 3 example tools that can be readily available and can be easily integrated into current workflows and the EHR. We noted variation in sentiment for all 3 tools in the care team design interviews. Patients also had varying levels of sentiment for each feature of the LPOC patient view. There were some features perceived as positive or slightly positive sentiment (eg, referrals and care team information). Participants did share their suggestions to improve specific features (eg, goals, active plans, SDoH Wheel, and medications) of the tools. The sentiments expressed in our study for CR and EpicCare Link indicate important considerations (eg, the need for a tool with clearer labels around goals and the status of these goals) for any digital tool (custom or Epic) that will need to be incorporated before successful adoption in practice. Given the varying levels of sentiment and participant recommendations, we determined that a customizable set of tools in Epic or a set of tools hosted through an independent digital platform for the care team and patients were 2 plausible options to meet user needs. Tailored health goals and customizable features on the digital solution align with current practices in the literature [48,49].

### Implications and Recommendations for Future Design and Practice

This study, to our knowledge, is among the first to examine the integration of SDoH screening into the clinical workflow from both the care team and patient perspectives. There is also limited prior work on the integration of social needs with other health-related needs to provide a more holistic picture of the patient.

Prior work has highlighted challenges in collecting and managing SDoH in real-world clinical workflows [4,23] and the variability in these workflows across settings [23]. Other research has explored Epic-enabled interventions. For example, Mazurenko et al [28] conducted usability interviews with emergency department care teams on the use of an Epic-integrated clinical decision support system to manage social needs, identifying similar requirements for user-friendly, customized views and the importance of timely data collection.

Beyond usability, there have been several larger-scale implementation studies. Ajibola et al [50] demonstrated that SDoH screening rates increased by 95% over 3 years across 3 community health centers using Epic with the Protocol for Responding to and Assessing Patients’ Assets, Risks, and Experiences tool; the authors reported, however, significant difficulties in capturing patient-specific details and ensuring effective connections to community resources—challenges similar to those reported in our study. Similarly, Rogers et al [51] described a custom Epic-integrated workflow linked to a community resource management platform, which screened over 111,000 Medicare and Medicaid patients and facilitated thousands of referrals, yet still required extensive workflow redesign and interdisciplinary coordination to be sustainable.

Some institutions have piloted more advanced solutions, such as Epic-based “smart referral” systems that trigger automated resource linkages based on screening results [52]. While promising, these efforts remain limited in scope and lack rigorous evaluation of downstream community linkages and long-term impact on patient outcomes. More recently, large-scale Epic database analyses (eg, Epic Cosmos) have underscored ongoing disparities in SDoH data completeness across racial and ethnic groups, reinforcing the structural challenges of standardization and equity in Epic-based SDoH initiatives [53].

Taken together, these studies highlight the growing movement to leverage Epic for SDoH screening but also reveal consistent challenges with usability, data completeness, workflow optimizations, and community resource integration. Our findings build on this literature by highlighting specific technological and workflow considerations, especially in the surgical setting, that are critical in the implementation of a custom or Epic-focused SDoH intervention. To enhance the digital tool, care team members recommended tailoring features (eg, care team, goals, and active plans) to the patient’s specific surgery, with additional detail provided for complex pathways such as cancer surgery. Both care team members and patients emphasized the need for clearer, nonclinical language and the inclusion of educational resources relevant to surgery type (eg, postsurgery diet). The tool should expand content on care teams, medications, and goals, with goals reworded and offering alternatives to sustain patient motivation. Usability could be improved by adding recent and future visits to the dashboard and by revising the SDoH section, offering both graphical and list-based options to accommodate different preferences.

Digital applications, if found to be effective and integrated into the clinical workflow, can be governed and maintained by specific AMC stakeholder groups, the AMC IT team, and the clinical department. We recognize that other settings such as community hospitals and community clinics have different governance structures and funding availability, which will be the subject of a subsequent study by our team. Additional investigation will also need to be considered about integration of applications across EHR platforms (using Substitutable Medical Applications, Reusable Technologies, and Fast Healthcare Interoperability Resources). A standard and robust platform requires extensive design and implementation consideration or trade-offs to account for standard data access and identity management, with the goal of broadening the reach of a potential application.

Based on our themes, participants (both care team members and patients) indicated a general receptiveness to using digital health tools, even among older patients. There is a growing body of evidence in the literature that indicates older adults increased the use of smartphones and other digital technology, especially during COVID-19 [54-56]. Our team also used multiple approaches to recruitment and interview sessions to mitigate against technological biases. There were, however, preferences around usability (eg, printing reports, large font size, and clear visuals) that indicated the need for desirable features to promote the use of digital health tools that are more approachable, possibly reflecting the needs of older patients. Some participants indicated the need for a nurse navigator to help better use the technology. Many participants also rely on caregivers who may be younger and receptive to digital health tools.

It is important to ascertain several of the mechanisms of action proposed by our intervention in this study. Our research team aims to achieve this by first conducting a pilot to evaluate the efficacy of the intervention regarding patient activation and its subcomponents (ie, confidence, self-management, and self-efficacy), as well as the constructs from SCT and GST and health-related needs. A future multiarmed effectiveness study can be conducted to further validate the intervention linkage with patient activation and subsequent effects on clinical end points such as readmission rates, emergency department visits, and surgical complications.

### Limitations

This study had several limitations. Our study was not linked to an evaluation of specific health outcomes [27]. Our formative interviews were conducted at outpatient clinics associated with our AMC; albeit, our AMC is one of the largest health systems in the Midwest and delivers complex surgery for a diverse pool of patients who live in the AMC city and across the state. We did, however, conduct convenience sampling for participants in our study, which may have impacted our formative interviews. By oversampling on racioethnicity and stratifying by neighborhood deprivation, we aimed to ensure that perspectives from historically marginalized populations were included. We acknowledge that this approach did not eliminate selection bias; however, it balanced feasibility with the need to capture critical variation across groups most relevant to disparities in surgical outcomes. Our goal was not to identify significant thematic differences by these factors, and such an analysis requires a different study design. We discussed our phase I and phase II findings with only the research team participants. Both care team members and patients in phase II were able to confirm and disconfirm many of our assumptions from our findings in phase I by their responses to the design considerations in phase II. This research should provide critical feedback that is less biased because we implicitly incorporated findings from phase I into our design activities, as opposed to asking participants explicitly to react to findings and obtain socially desirable responses. In addition, some patients may not have fully understood what health-related needs were during interviews, which could have impacted our results; however, our team followed systematic procedures to ensure that patients were made aware of the concepts covered in our interview sessions, and we documented challenges around awareness as well. More research needs to be conducted to further validate and expand on the themes identified in our study, including the use of novel participant engagement strategies among vulnerable populations for research on sensitive topics. It is particularly important to build trust and engagement among many of the communities represented in our study because they have been historically overlooked in research and may experience higher levels of skepticism about the intent of our research and its capacity to make a difference in their respective communities.

### Conclusions

In this study, we obtained qualitative feedback from both care team members and patients in our AMC’s department of surgery on how to collect and manage health-related needs, including social needs. We conducted thematic analysis to identify convergent and divergent themes about critical gaps and potential solutions. Health-related needs collection and resolution processes varied greatly between the perspectives of both care team members and patients. Both patients and care team members frequently mentioned social support and home care as their top social need. Based on the formative interviews, we selected digital tools from Epic as potential digital solutions to present to a subset of our participants. We identified a desired set of features in an ideal digital solution based on our sentiment analysis of the design sessions. Further research is necessary to continue improving our proposed solution to promote health-related needs screening and management for patients who undergo complex surgery.

## Supplementary material

10.2196/77995 Multimedia Appendix 1 Patient interview guides.

10.2196/77995 Multimedia Appendix 2 Care team interview guides.

10.2196/77995 Multimedia Appendix 3 COREQ guidelines for research study on exploring a digital health solution to collect and manage health-related needs for patients who undergo complex surgery.

10.2196/77995 Multimedia Appendix 4 Patient activation view.

10.2196/77995 Multimedia Appendix 5 Patient and care team current needs tables.

## Abbreviations

AMC: academic medical center
CBO: community-based organization
COREQ: Consolidated Criteria for Reporting Qualitative Research
CR: Compass Rose
EHR: electronic health record
GST: goal-setting theory
HIPAA: Health Insurance Portability and Accountability Act
ICD-10-PCS: International Classification of Diseases, Tenth Revision, Procedural Codes
ICD-9-CM: International Classification of Diseases, Ninth Revision, Clinical Modification
LPOC: Longitudinal Plan of Care
OOI: Ohio Opportunity Index
SCT: social cognitive theory
SDoH: social determinants of health
SMART: specific, measurable, achievable, relevant, and time-bound
