# Supplementary figures and images for: Exploring a Digital Health Solution to Collect and Manage Health-Related Needs for Patients Who Undergo Complex Surgery: Mixed Methods Study
Source: J Med Internet Res. 2025 Dec 8;27:e77995. doi: 10.2196/77995 (PMC12684001; doi:10.2196/77995)

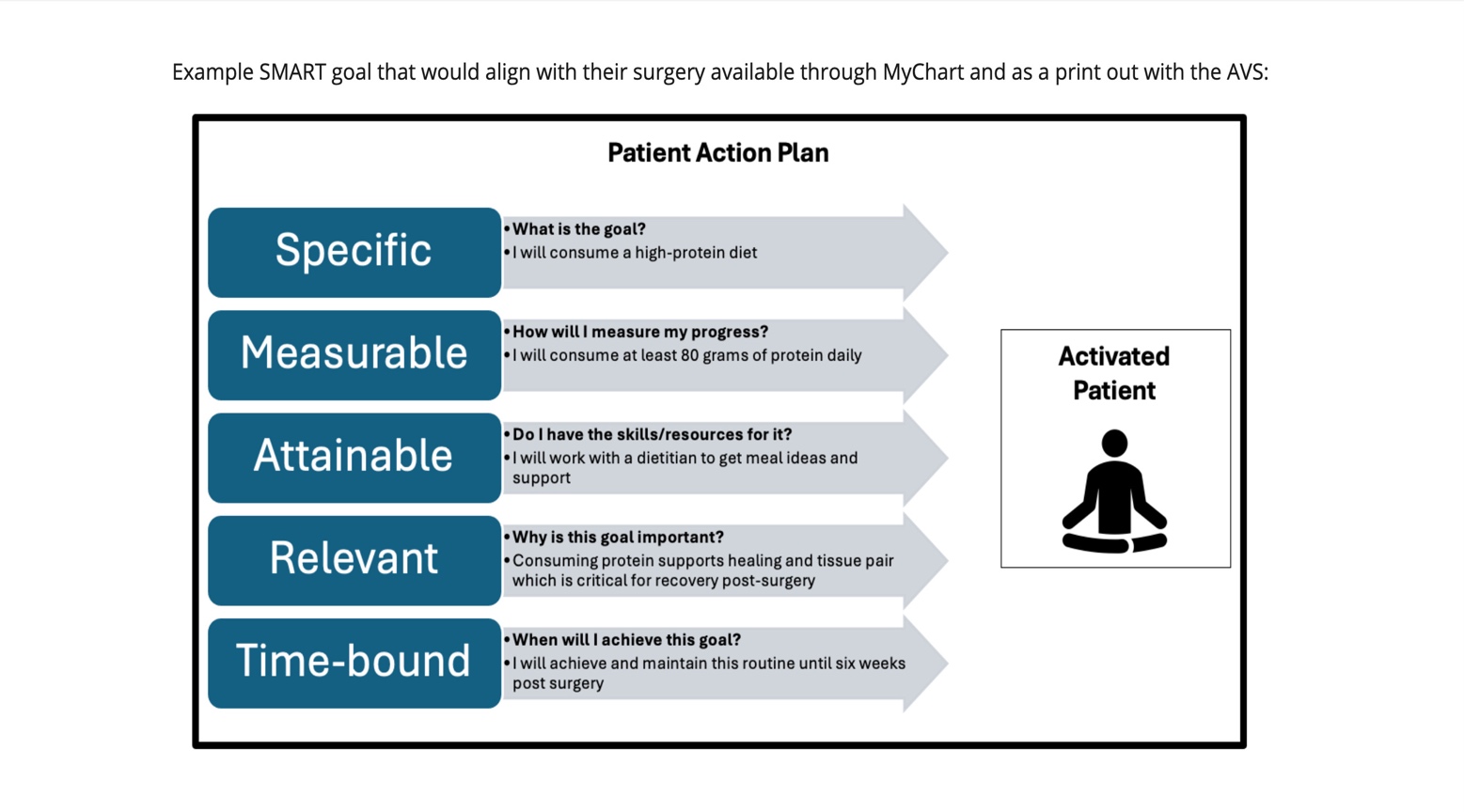

Supplement: Multimedia Appendix 4 [file jmir-v27-e77995-s004.docx]
